# The Power of Shape: How Shape of Node-Link Diagrams Impacts Aesthetic Appreciation and Triggers Interest

**DOI:** 10.1177/2041669518796851

**Published:** 2018-09-08

**Authors:** Claus-Christian Carbon, Tamara Mchedlidze, Marius Hans Raab, Hannes Wächter

**Affiliations:** Department of General Psychology and Methodology, University of Bamberg, Germany; Research Group EPÆG (Ergonomics, Psychological Æsthetics, Gestalt), Bamberg, Germany; Bamberg Graduate School of Affective and Cognitive Sciences, Germany; Institute for Theoretical Informatics, Karlsruhe Institute of Technology, Germany; Department of General Psychology and Methodology, University of Bamberg, Germany; Research Group EPÆG (Ergonomics, Psychological Æsthetics, Gestalt), Bamberg, Germany; Institute for Theoretical Informatics, Karlsruhe Institute of Technology, Germany

**Keywords:** empirical aesthetics, node-link diagrams, application, information visualization, curvature, form, shape, interest, preference, beauty

## Abstract

Positive effects of aesthetically appreciated designs have long been studied and confirmed since the 19th century: such designs are more enjoyable, they are more forgivable for glitches and can increase users’ performance. In the field of information visualization, studies of aesthetics are still a niche approach. In the current study, we aim to specifically understand which parameters in a visualization of node-link diagrams make them aesthetically pleasing-an important extension to already existing research on usability and readability aspects. We investigated how the shape of the outline of such diagrams influences the aesthetic judgments on two of the most important dimensions of aesthetic appeal: beauty and interest. We employed different outlines to node-link diagrams and compared them with uniformly filled shapes, varying two important variables typically impacting aesthetics: complexity and curvature. This was done for a short (100 ms) and ad libitum presentation time. Diagrams with curvier outlines were perceived as more beautiful, while diagrams with more complex outlines were considered to be more interesting. These dependencies already exist for presaccadic perception (100 ms) and are slightly stronger for unlimited presentation time. We also found that curvature is a predictor for beauty only for unlimited presentation time. Aesthetic appeal was very similar for diagrams and pure shapes, so many results from fundamental research on aesthetics can potentially be transferred to the community of network visualization, assisting to improve visualizations also in aesthetic regards.

## Introduction

Aesthetics can be defined as “a concept that relates to the beauty in both nature and art, as something that enlivens or invigorates both body and mind, awakening our senses” (Cawthon & Moere, 2007, p. 1). It can also be defined in a very much broader sense to underline the relevance of aesthetics for a series of cognitive and affective processes: “To understand what people appreciate, like, love, or prefer, and why they do so is of essential relevance for everyday life events where a clear rational basis for decision making is often not available” (Carbon & Jakesch, 2013, p. 2123).

Individuals from various disciplines have tried to understand the notion of aesthetics by applying the tools available to them: Philosophers tried to explain it using human language, artists investigated the meaning and the boundaries of aesthetics by producing pieces of art and exposing them to human judgment, cognitive psychologists explored it by applying statistical analysis to the results of controlled user studies measuring human responses, and mathematicians tried to measure it defining a plethora of analytic functions.

It is not surprising that in the field of *Information Visualization* (InfoVis—the study of visual representations of abstract data to be optimally processed by humans), which deals with visual representations of data, the investigation of aesthetics to increase insight was listed as one of the ten most important open problems: “Engineering aesthetics into information visualization remains a challenge” (Chen, 2005, p. 15). The importance of this problem is explained by previous investigations indicating that aesthetics can positively affect apparent and actual usability (Kurosu & Kashimura, 1995; Norman, 2002; Tractinsky, Katz, & Ikar, 2000) and is, more generally, related to positive mental states, promoting problem-solving abilities (Fredrickson, 1998; Isen, Daubman, & Nowicki, 1987). Aesthetics plays a significant role in a system’s overall attractiveness (Carbon & Leder, 2005; Tractinsky, Cokhavi, & Kirschenbaum, 2004) as a significant incentive for initial use and beyond—it gives pleasure to the user (Biederman & Vessel, 2006)!

**Figure 1.**
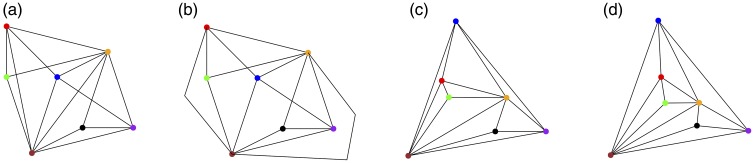
An example of optimization of various metrics for a construction of a readable node-link diagram: (a) An arbitrary node-link diagram, (b) a different node-link diagram of the same graph, where two edges are redrawn using bent polylines to reduce the number of crossings, (c) the nodes are repositioned to achieve a straight-line drawing without crossings, and (d) the nodes are repositioned to make the angles around nodes as even as possible.

In the field of Graph Drawing, the term “aesthetics” has been heavily used; however, “aesthetics” refers there to metrics on node-link diagrams that intuitively determine their usability and readability (Kaufmann & Wagner, 2001), rather than to the aesthetic quality of their appearance. Examples of such metrics are, inter alia, number of edge^1^ crossings, number of edge bends, and angular resolution (refer to Figure 1). The connection of those metrics to user performance has been studied empirically (e.g., Huang, Hong, & Eades, 2006; Huang, Huang, & Lin, 2016; Purchase, 1997; Purchase, Hamer, Nollenburg, & Kobourov, 2012; Ware, Purchase, Colpoys, & McGill, 2002). Node-link diagrams have been empirically studied from the perspective of enjoyment (Saket, Scheidegger, & Kobourov, 2016) and subjective usability prospects (Huang et al., 2006; Purchase, Allder, & Carrington, 2002; Purchase, Hamer, et al., 2012). However, the extent to which these metrics determine the aesthetics in the sense of perceived beauty of node-link diagrams is far from being understood.

In several experiments (Dwyer et al., 2009; Purchase, Pilcher, & Plimmer, 2012; van Ham & Rogowitz, 2008), researchers asked participants to create node-link diagrams themselves by using various interactive environments which allowed for operations of node movements and edge modifications. The studies similarly showed that users tend to minimize edge crossings. Some other evident properties that participants payed attention to were: revealing the graph clustering, and in opposite, maintaining similar distances between nodes, enclosing clusters within bounding hulls, symmetrizing, keeping the edges short, and placing the node with the highest degree centrally or above.

### The Present Study

Motivated by the finding of van Ham and Rogowitz (2008) that observers construe a bounding hull to “visually delineate” (p. 1339) a cluster of nodes in a diagram, we asked ourselves whether such a simple characteristic like the shape of a node diagram’s outline might be a parameter of aesthetic appeal worthwhile to study. To dig into the core of this question, in the present study, we only look at the outline of the whole node-link diagram rather on outlines of the individual clusters. This focus is grounded in Gestalt theory of perception: Boundary edges and contours contribute to the separation of figure from the ground (see also Wong & Sun, 2006) and thus are among the most important distinctive features that make a graph a distinctive, self-contained object.

Since people generally prefer and enjoy objects that seem beautiful to them (Norman, 2002), we investigated, first of all, the subjective ratings of **beauty** of node-link diagrams. Beauty is probably “the” most important variable in aesthetics all along, starting in philosophy of aesthetics (e.g., Kant, 1790/1922) up to very recent psychological theories (e.g., Reber, Schwarz, & Winkielman, 2004)—it is also the concept mostly referred to when people have to judge the aesthetics of objects (Jacobsen, Buchta, Köhler, & Schroger, 2004) or when they are asked to describe artworks (Augustin, Carbon, & Wagemans, 2012) or design products such as cars or clothing (Augustin, Wagemans, & Carbon, 2012). It is also known that beauty alone is not enough to keep the viewers investigating a visual stimulus (Silvia, 2005).

There is actually evidence that **interest**, as a variable known to indicate potential longer termed engagement with respective stimuli, predates moments of insight, that is, experiences of suddenly understanding a complex visual structure (Muth, Raab, & Carbon, 2015). Thus, alongside with beauty, we followed the idea of Silvia (2005) and investigated the interest triggered by the stimuli. There is an analog to these ideas from the area of display design, where it is recommended to create displays that at first glance are aesthetically interesting and convey information on closer examination (Fogarty, Forlizzi, & Hudson, 2001).

**Curvature** has been identified as a typical physical predictor of beauty and is being preferred against angularity in many object classes (for an overview Gomez-Puerto, Munar, & Nadal, 2016), for instance concrete consumer products (Westerman et al., 2012), car interiors (Leder & Carbon, 2005) or car exteriors (Carbon, 2010), but also for meaningless or abstracted material such as polygons (Silvia & Barona, 2009)—Bar and Neta (2006) even claimed quite generally “Humans prefer curved visual objects”, a finding which was also replicated in the haptic domain (see Carbon & Jakesch, 2013; Jakesch & Carbon, 2011). This general preference is not without doubt, especially when specific designs are susceptible to Zeitgeist effects (Carbon, 2010), which however seem to play no essential role in the present study addressing graphs and mere shapes with no direct connection to products or objects. General positive associative factors with curved shapes documented by Palumbo, Ruta, and Bertamini (2015) seem to be much more important in the present study, so we included curvature as a possible predictor for beauty.

**Complexity** is another prominent candidate for modulating preferences. There are indications that complexity is a good predictor for interest (see Muth et al., 2015). How complexity is exactly related to beauty remains unsure as some claim parabolic functions with medium complex stimuli showing the highest hedonic value (Berlyne, 1970, 1971)—a construct strongly related to beauty—, while others have empirically revealed simple linear relationships (e.g., Nadal, Munar, Marty, & Cela-Conde, 2010); very recent research even challenges any of these strict views, as large individual differences of the link between complexity and preferences have been documented ever since (Gucluturk, Jacobs, & van Lier, 2016). Different ranges or distributions of complexity across employed stimuli might be the cause of this contradiction. The usage of different stimulus classes across studies could be another factor for this discrepancy, as each class of stimuli might have a specific maximum of complexity. In a recent study, Adkins and Norman (2016) studied snowflake silhouettes and computer-generated solid objects of various complexity and observed that the most and least complex objects were perceived as being the most beautiful, while for the snowflakes, the results were more uniform. Actually, most participants perceived only the complex snowflakes as very beautiful.

**Figure 2.**
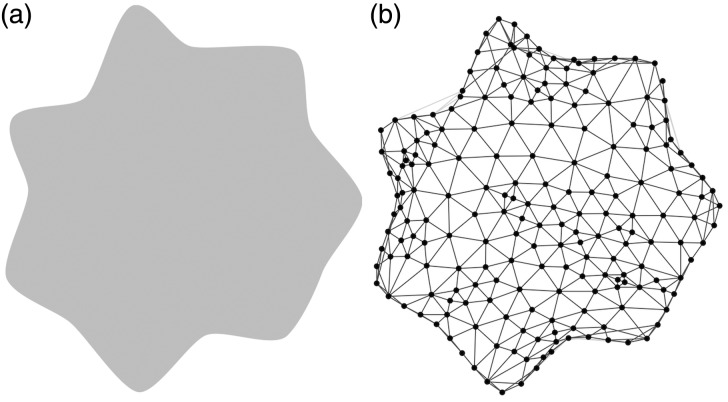
Example of a stimulus: (a) a shape and (b) a node-link diagram using the shape as an outline.

We preselected a set of shapes that vary in their complexity and curvature and used these shapes as outlines for node-link diagrams. We controlled the appearance of the node-link diagrams by only changing their boundaries and by keeping the drawings of the actual graphs similar. This way we aim to find out whether the perceived beauty of the visualization can be influenced without changing the values of visualization metrics that possibly affect the usability of these visualizations. To understand whether the beauty of the node-link diagrams can be predicted by the shape of their outlines, we conducted the experiment both for node-link diagrams and for pure shapes (Figure 2). To further test whether the outline of the node-link diagrams contribute the first impression they make, we investigated all research questions with a short, presaccadic presentation time (PT = 100 ms) and compared the results to a presentation with unlimited time.

## Study

### Method

#### Rationale

Our first goal was to investigate whether the curvature and the complexity of the outlines of node-link diagrams influence the perceived beauty and trigger interest. An existence of such an influence can give hints to the designers of node-link diagrams on how to attract a viewer’s attention to investigate the node-link diagram.

Our second goal was to understand whether the beauty and the interest ratings are similar for the shapes and for the node-link diagrams using those shapes as outlines. This is an important question, as we know much more about the beauty of shapes than about the beauty of node-link diagrams. A positive answer to this question would give us hints on what node-link diagrams should look like to be attractive to humans.

Our third goal was to understand whether the very first impression of a node-link diagram’s beauty is different from a judgment after a more thorough visual inspection. To investigate this question, we utilize Norman’s (2005) simple human perception model which distinguishes three levels of perception: (a) The visceral level is responsible for the initial judgment of things, our intuitive reactions, for example, responsible for identification of dangerous situations. (b) The behavioral level is responsible for automated routines of perception and action, for example, playing piano and driving a car. (c) The reflective level is responsible for conscious thinking and access of memories. To capture the intuitive responses of the participants, staying closer to the visceral level of perception, we exposed the participants to the stimuli for 100 ms, a time interval before the first saccade, the first eye movement away from the initial fixation point, usually takes place. We compared this to a second condition, where—ceteris paribus—the participants are exposed to the same stimuli for unlimited time, which we consider representative for a more behavioral mode of perception *sensu* Norman.

#### Procedure

**Figure 3.**
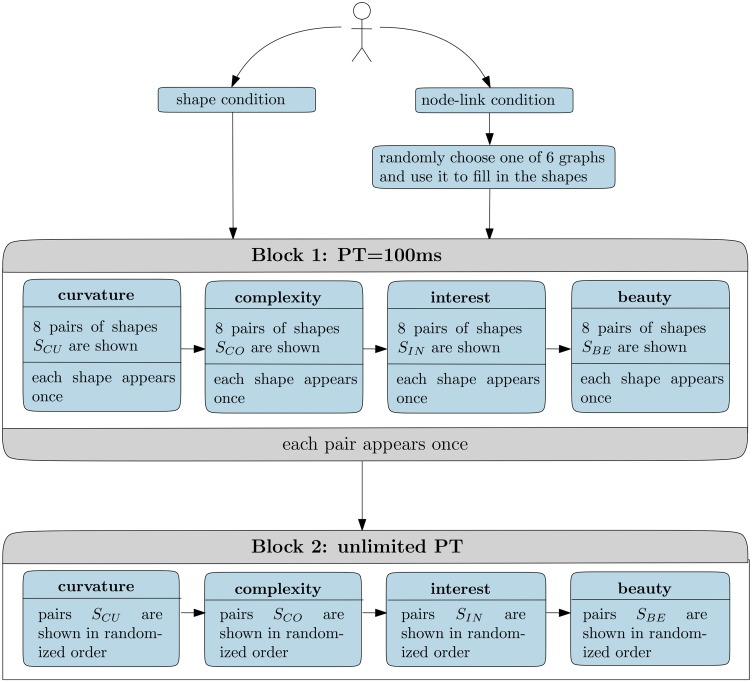
Course of the experiment. Pairs of stimuli shown in the subblocks for curvature, complexity, interest, and beauty are denoted by *S_CU_*,* S_CO_*, *S_IN_*, and *S_BE_*, respectively. PT = presentation time.

Figure 3 shows a brief illustration of the experimental design. To avoid a carry-over effect (assessments for node-link diagrams influencing the shape assessments and vice versa), we randomly assigned participants to two different groups, one exposed to shapes, and the other exposed to node-link diagrams. In the following, we describe the design of the experiment for the shapes. The procedure with node-link diagrams is identical, with the only difference that instead of shapes, node-link diagrams of graphs using that shape as outline were employed. We tried to keep the study short (∼10 min), to avoid boredom of the participants and therefore lack of concentration.

The study was divided into two blocks: In the first block, the PT for stimuli was 100 ms,^2^ whereas in the second block, the stimuli remained on screen until key press. Each block comprised four subblocks where we asked for participants’ assessments stimuli regarding *curvature*, *complexity*, *interest*, and *beauty*, respectively. The order of subblocks was fixed. Curvature and complexity were regarded as independent “object-specific” variables, whereas beauty and interest were treated as dependent variables.

Since perceptual differences between stimuli are in many cases quite small, we looked for a measurement that is sensitive enough to uncover such subtleties. The typical employed rating scale method is a very efficient but not the most sensitive method to capture preference assessments. In contrast, paired comparisons offer much better sensitivity than rating scales in detecting differences (see Eisenberg & Dirks, 1995), so we employed such a high-sensitive pairwise comparison task, especially as we used a PT limitation paradigm. The participants were asked to select whether the left or right stimulus was “clearly more” (=3), “more” (=2), or “weakly more” (=1), for example, curvy (or: complex, interesting, beautiful, respectively) than the right or left stimulus, respectively.

Every subblock (e.g., Subblock 1 *curvature*) consisted of eight trials, and every trial presented two distinct stimuli to the viewer side by side. We kept possible preference biases based on the mere-exposure effect (Zajonc, 2001) constant across stimuli by means of counterbalancing. In particular, each stimulus was presented only once within a subblock, and therefore each stimulus is seen by the viewer exactly eight times (two PTs by four subblocks). For each subblock, the eight pairs were randomly drawn from the set of all possible 120 pairs (refer to *stimuli* section), and the chosen pairs were not considered for the selection in the following subblocks. Overall, *k* = 4 × 8 = 32 distinct pairs of stimuli were shown in the first block. In the second block, the same 32 pairs were presented, but the order of trials was randomized anew within each subblock.

The study was implemented on an online platform developed based on Django framework (https://www.djangoproject.com)—a free and open-source web framework, written in Python. The procedure started with a welcome message and a brief introduction to the experiment. Participants were requested to spontaneously select which stimulus showed the regarding variable more than the other stimulus of the pair. For instance, in the first subblock (curvature), they were asked “Which visual object appears more *round* to you?” The regarding questions of the subsequent blocks were about which object was more *complex*, more *interesting*, and more *beautiful*, respectively. Note: we used “round” instead of “curved,” as our pilot studies indicated that participants had a better intuitive understanding of an item that is “round” instead of “curved” (see Carbon, 2010).

**Figure 4.**
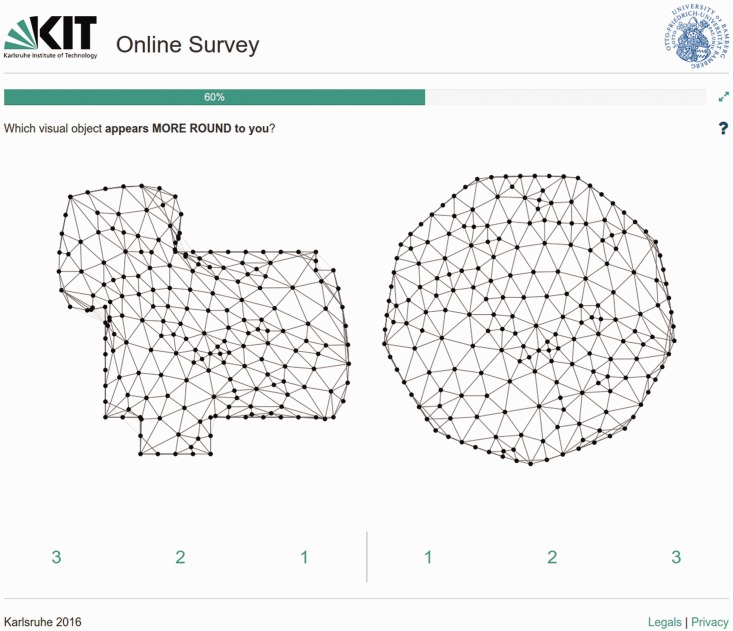
Screenshot from a trial within the second block.

In the first block, each trial started with a centered fixation cross (1000 ms)—for the unlimited PT in the second block, this fixation cross was not present to make implicitly clear that fast reactions were not demanded for this part of the experiment. Subsequently, a pair of stimuli was presented side by side (Figure 4) for 100 ms (first block) or as long as the participant answered the regarding question (second block). In the first block, we masked the display subsequently with a backward mask of white noise for 100 ms, in order to minimize visual persistence beyond the time of presentation. The next trial started as soon as the participant entered an answer by pushing a target button. The whole study took on average 10 minutes per person.

#### Stimuli

**Figure 5.**
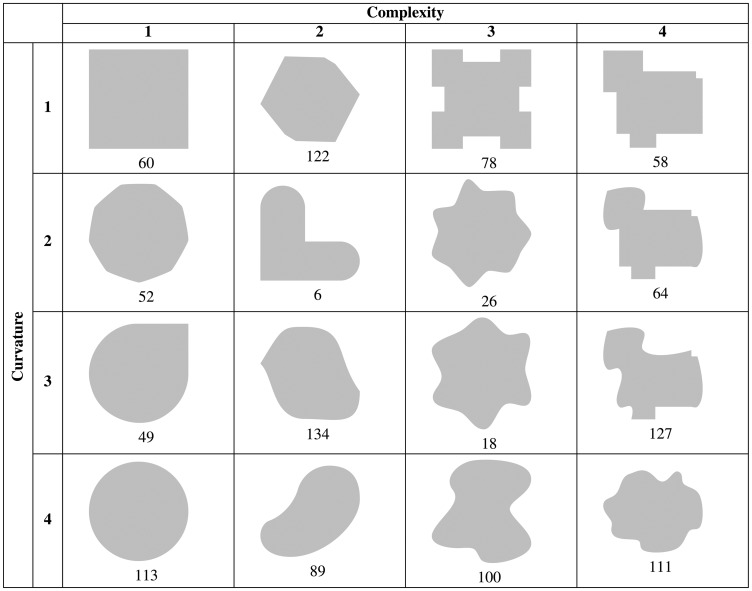
Finally, selected shapes for the main study (with original numbering of the stimuli originated from the pilot study).

In a preliminary step, we created 138 shapes with different appearances regarding curvature and complexity, as diverse as possible. With an internal expert pilot study with five raters, we got ratings for curvature and complexity for all shapes. We calculated the mode for each shape and both properties to select a representative shape (best fit) for each of the 16 curvature or complexity combinations, since our goal was to investigate how beauty and interest are explained by curvature and complexity. Fleiss’ kappa (κ) test, a statistical measure for calculating the reliability of agreement between raters, revealed “substantial” interrater agreement (according to Landis & Koch, 1977) for the selected shapes: κ = 0.68 for curvature and κ = 0.72 for complexity. See Figure 5 for the finally preselected *shape stimuli*.

On basis of those selected shapes, we constructed node-link diagrams that employ exactly the respective shapes as outlines. As graphs, we used six randomly generated Delaunay triangulations with 200 nodes. These graphs generally have long boundaries, a property that was required to realize sufficiently complex outlines.

**Figure 6.**
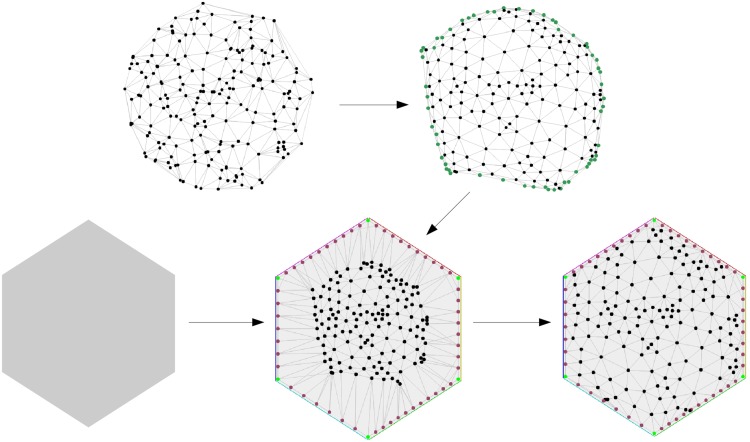
Depiction of the algorithmic procedure to create the node-link stimuli. Nodes to be placed on the boundary are identified (top-right) and placed on the boundary (bottom-middle), force-directed algorithm is run that preserves the position of the nodes on the boundary (bottom-right).

For constructing a node-link diagram of a graph having a given shape as outline, we used a force-directed approach (Jacomy, Venturini, Heymann, & Bastian, 2014)—an algorithm that simulates a physical system where the nodes are physical bodies, that attract or repel each other dependent of whether the nodes are connected by an edge or not. This physical system is let go until it achieves an equilibrium. See Figure 6 for a depiction of the algorithmic procedure to create the node-link stimuli.

Initially, a planar layout was constructed, and the nodes that were suitable to lay on the boundary were detected (for the pipeline of the algorithmic procedure, refer to Figure 6). These are not exactly the boundary nodes, since those are usually not enough to mimic the outline of the given shape. These nodes are distributed on the outline of the given shape. The remaining nodes are put inside the outline, and the force-directed method is run, by keeping the positions of the outer nodes fixed. Finally, the edges that connect two points of the outline and do not lie inside the shape are colored gray, to achieve the similarity with the given shape. Note: Our node-link diagrams are visualizing six planar graphs with very specific properties. By using polyline or curvilinear edges, a similar drawing algorithm could be applied to all planar graphs, and the overall appearance of the graph is similar. However, the results have to be generalized with caution for nonplanar graphs, specifically with many crossings.

For the *k* = (16 × 15)/2 = 120 possible pairs of shape stimuli, we generated two images, for two possible selections of the shape placed on the left. For each shape stimulus and each of the six graphs, we constructed the corresponding *graph stimulus*, which showed the selected graph within the outline represented by the shape.

#### Participants

To get participants from various backgrounds, we announced the survey on different public websites like Reddit^3^ and Facebook.^4^ As incentive, five Amazon vouchers worth EUR 25 each were raffled among the participants that were willing to take part in the lottery and provided us their e-mail address. Those addresses were stored separately from the remaining data to protect privacy.

Altogether *N* = 233 people participated in the online study, from which 111 were assigned to shape and 122 to graph conditions with average age 29.4 years and 30.9 years, respectively. With respect to gender, 117 were female, 103 male, and 13 stated *other*. The origin of the participants was mainly from Germany (71.0%), followed by users from United States (12.5%), Great Britain (2.6%), Austria (2.2%), and 18 other countries. Regarding the education, 6.0% had a PhD, 33.1% a master or diploma degree, 27.0% a bachelor degree, and 32.6% indicated high school as highest gained level of education—1.3% declared having no education of the asked categories.

Note: The sample employed in our study was restricted to people with Internet access and is biased toward people who consume the websites that we used for recruitment or who were associated with the authors’ circle of acquaintances. No other confounding was evident.

### Results and Discussion

We incorporated all participants’ data in the following analyses. Since the data are based on pairwise comparisons, we obtained one explicit rating that we further processed in the following way: a positive rating for the chosen object and a negative rating with same absolute value for the other. Shapiro–Wilk tests indicated that the data were not statistically deviant from being normally distributed.

When time was not restricted, participants took about 3 s to evaluate a graph or a shape, which indicates that judgments were not just visceral reactions (in Norman’s sense), but were processed with conscious awareness (behavioral level). The mean response time in the unlimited conditions was 2907.1 ms for graph and 2763.9 ms for shape conditions.

**Figure 7.**
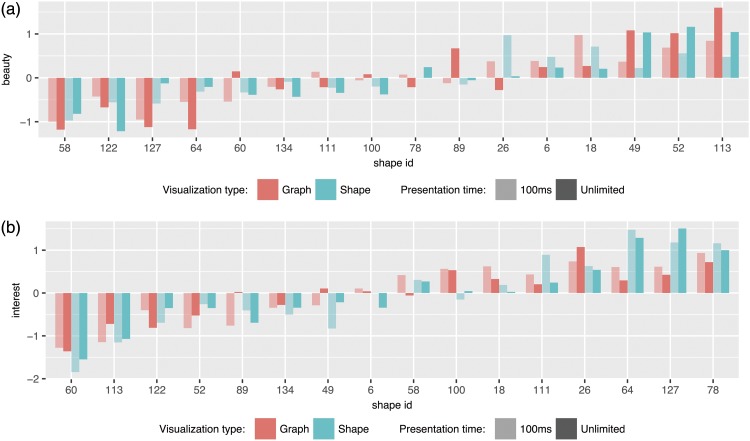
Mean values of (a) beauty and (b) interest for the 2 × 2 = 4 experimental conditions, sorted by their overall mean.

To obtain an overview of how the ratings of beauty and interest vary over the two presentation types (shapes vs. graph) and the two PTs, we plotted the two dependent measures beauty and interest for all 2 × 2 = 4 experimental conditions (Figure 7). Our first perceptual inspection of the data made clear that we revealed a generally high similarity of ratings across experimental conditions.

A statistical analysis of the correlations between the dependent variables for both PTs and for all collected variables showed, however, that beauty assessments were seemingly based on different features or processes when facing limited (100 ms) compared with ad libitum PT, *R*(16) = .780, *p* = .0004. For interest, we revealed much closer relationship between both PT conditions, *R*(16) = .871, *p* < .0001. The high, but still far from perfect relationships between PT conditions, point to potential “microgenetic processes” (Bachmann, 2000) which have already been progressed quite far when a stimulus of the used kind is presented for only 100 ms, but where further processing is still going on (Carbon, 2011). Interestingly, such an ongoing microgenesis was not detectable for the predictors complexity and curvature, which showed nearly perfectly identical assessments at a PT of 100 ms and also in the case of unlimited PT, that is, *R*(16) = .960, *p* < .0001 and *R*(16) = .978, *p* < .0001, respectively. This indicates that complexity and curvature assessments do not require very deep cognitive processing as they are reliably processed even under very limited presentation conditions. As such, both factors might be interpreted more like content factors than style factors following the style-follows-content hypothesis of microgenesis in scene perception (Augustin, Defranceschi, Fuchs, Carbon, & Hutzler, 2011; Augustin, Leder, Hutzler, & Carbon, 2008).

**Figure 8.**
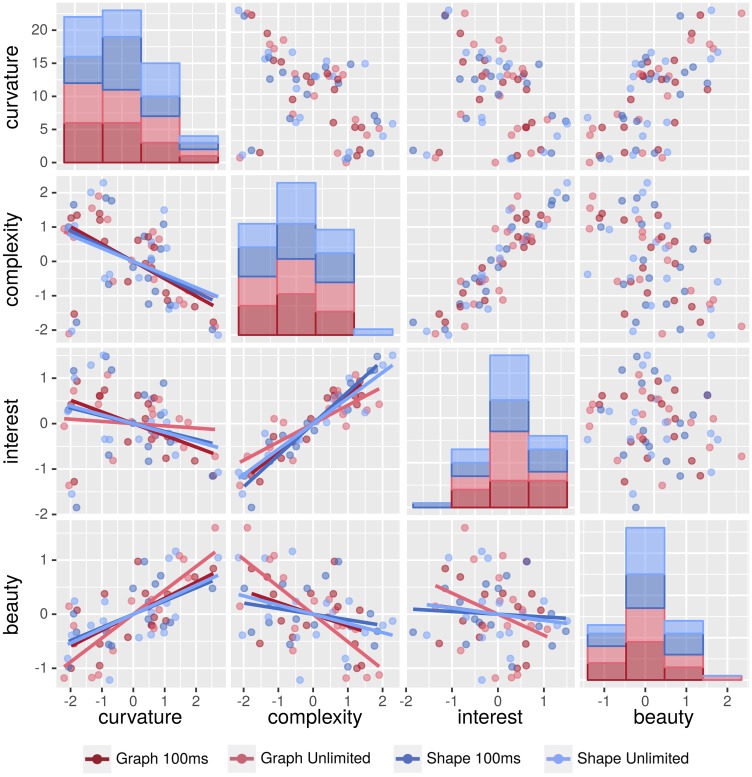
Scatter plots for all four variables, including linear regression lines and distributions of ratings. The 4-by-4 plot panel is organized the following way: the top-left to bottom-right diagonal shows the distribution of ratings for curvature, complexity, interest, and beauty, respectively. The upper triangle shows the regarding bivariate relationships, and the lower triangle additionally provides the linear regressions. All data are split by the conditions *presentation type* (graph vs. shape) and *presentation time* (100 ms vs. unlimited).

As we were particularly interested in how beauty and interest can be predicted taking our independent variables curvature and complexity simultaneously as predictors, we furthermore conducted two separate linear regression analyses for each PT condition. Refer also to Figure 8 for the scatter plots of all four variables plus their interrelationships.

As indicated in Table 1, we obtained high rates of explained variances in general.

**Table 1.** Results of the Linear Regression Analyses.

Presentation time	Dependent variable	Reference	Outcome	*p*
100 ms	Beauty	**model**	***R*** **^2^** **^ ^= .455**	**.0193**
		complexity	*B* = .024	.8697 *ns*
		**curvature**	***B* = .308**	**.0193**
100 ms	Interest	**model**	***R*** **^2^** **^ ^= .900**	**<.0001**
		**complexity**	***B* = .691**	**<.0001**
		curvature	*B* = .091	.1530 *ns*
Unlimited	Beauty	**model**	***R*** **^2^** **^ ^= .825**	**<.0001**
		**complexity**	***B* = −.328**	**.0026**
		**curvature**	***B* = .313**	**.0009*.***
Unlimited	Interest	**model**	***R*** **^2^** **^ ^= .722**	**<.0001**
		**complexity**	***B* = .485**	**<.0001**
		curvature	*B* = .141	.0629 *ns*

*Note. ns* = not significant. Bold face is used to indicate significant effects.

Beauty was mainly based on curvature aspects, at 100 ms actually exclusively on curvature. When PT was not limited, also complexity was taken into account, but here with a negative relationship: the more complex the stimulus, the less participants liked it. With curvature, it was quite similar across PT conditions: The rounder the stimulus was assessed, the higher it was assessed regarding beauty—a shape that is very near to a circular form was appreciated the most. Actually, reports of preferences for round shapes are quite numerous (see Gomez-Puerto et al., 2016), so these results were fully expected and reflect typical data from more complex domains of everyday objects (Westerman et al., 2012), including other modalities such as the haptic sense (Jakesch & Carbon, 2011).

For interest, we gained a very different data pattern: Here the essential factor was always complexity, with more complex stimuli being assessed more interesting—a finding that is compatible with other domains of visual stimuli such as artworks (Friston, Thornton, & Clark, 2012; Muth et al., 2015).

## General Discussion

We investigated the role of curvature and complexity of the outlines of node-link diagrams as drivers for generating interest and aesthetic appeal for the users of such types of graphical information. Therefore, we let 233 persons evaluate node-link diagrams with various outlines as well as shapes comprised by those outlines.

We found that curvature is a powerful predictor for beauty, but complexity has also its role when a beholder can inspect the stimulus longer—interestingly, our participants did prefer less complex stimuli under unlimited PT conditions regarding beauty. This supports the findings by Purchase, Hamer, et al. (2012) that node-link diagrams with circular-arc edges are liked more than straight line drawings. Taken together, this indicates that such aesthetic principles, which were often shown for artworks and consumer products, play also a role for visualization of information, for instance, in node-link diagrams.

For being interesting, most importantly, the stimuli have to be more complex—curvature was not important for being assessed as interesting. This makes clear that interest is not just a facet of beauty but has its own rules. As interest is an important variable for inducing longer termed attention to a stimulus, this is very important to know.

We found very similar data pattern for shapes and graphs drawn using those shapes as outlines, so the very general rule is that the outline should be optimized with priority, especially if it is about optimizing the beauty of a graph.

The differences found between PTs for the employed stimuli point at a difference in the conception of beauty that might be explained by a microgenetic view (Bachmann, 2000): The impact of the outline’s curvature is greater at second glance, but interest is crucial from the very early beginning and mainly triggered by complexity. This is in line with the idea by Muth et al. (2015) that complex visual structures can be perceived as a promise for an aesthetic riddle. Complexity might just be a good predictor for the degree of information density and so triggers attention, a cognitive construct that is very much linked with interest.

This microgenetic view (i.e., the idea that the final “percept”—what we eventually perceive—is generated by subsequent perceptual subprocesses, see Carbon [2011]) on the dynamics of appreciation, has already been established for works of art (Augustin et al., 2008, 2011) and does seem to be an interesting avenue for future research. The difference in model fit we have observed here should be taken as a starting point to target the dynamics of perception unfolding over the first few hundreds of milliseconds. Those dynamics of unfolding appreciation might have practical implications in situations where information is competing for observers’ attention. Graphs that are appealing at the very first glance might be the ones that get more attention (or even any attention at all).

So, there is merit in transferring aesthetic principles into the domain of information visualizations. We have shown that node-edge-structures without semantics, that is, syntactically well-formed node-graphs generated by random data, are appreciated in a way we might compare to the appreciation of artworks. There are two promising directions from here:

1. Expand the syntactical approach (as we have focused on the graphs’ outlines here; one might think of regarding shape and length of edges).
2. Start to introduce meaning by depicting real-world data (which would allow evaluating if more beautiful graphs are also more understandable).

Our node-link diagrams were generated by an algorithm and did not allow for any deeper insight. A further study should address riddle-solving aspects of appreciation by using node-link diagrams bearing semantic information (see point 2). This would, in Norman’s (2005) terms, add the (more) reflective level and allow for a closer look at the interaction of visual and semantic properties.

## Notes

1 A graph usually consists of several nodes (*aka* vertices), where a node represents a data point, that are usually depicted by dots or circles. Edges are nondirectional connections between such nodes, representing some kind of relation. A node does not have to have any edge connections, and the number of edges a single node is connected to is not limited. However, any edge must be connected to a node at both ends. Beyond these syntactic rules, any meaning (What is represented by a node? What kind of relation is implied by an edge?) is domain-specific. For a given graph (i.e., fixed node-edge-system), the challenge is to project it onto a two-dimensional plane in a way that structure and meaning are conveyed to the spectator. As edge length is arbitrary, there are in fact no syntactic restrictions for node placement.

2 The experiment was deployed as an online test. To ensure a presaccadic presentation time, stimulus presentation including the following mask was embedded in a video that was played back using HTML5 (with MP4 and webM files to maximize compatibility). To be sure that the timing was accurate, we conducted an extra timing study where we generated black-white-black videos with a 100ms whitescreen using the same algorithms that were used for the stimulus videos. The actual whitescreen duration was measured with a photodiode and an oscilloscope for a standard Android phone, and with a standard desktop PC running Windows 10 for both IE11 and Mozilla Firefox (for each device, we measured the MP4 as well as the webM video playing on the website), and an older netbook running Win XP (here: webM only as no MP4 codec was available). All measurements indicated presentation times of 100 ms ± 5 ms.

3 https://www.reddit.com is a social news aggregator, and https://www.reddit.com/r/SampleSize/ is a specific page for recruiting participants in online studies. It has currently more than 35,000 subscribers.

4 https://www.facebook.com/ is a social media platform.
